# Capture myopathy in a wild Clymene dolphin (*Stenella clymene*) stranded alive on the coast of Ceará State, Brazil

**DOI:** 10.29374/2527-2179.bjvm00625

**Published:** 2025-05-08

**Authors:** Vitor Luz Carvalho, Daniel Araújo Viana, Alexsandro Antonio Portilho Damasceno, Maria Vivina Barros Monteiro, Frederico Ozanan Barros Monteiro

**Affiliations:** 1 Associação para Pesquisa e Preservação de Ecossistemas Aquáticos – Aquasis, Caucaia, CE, Brazil; 2 Universidade Estadual do Ceará, Fortaleza, CE, Brazil.; 3 Programa de Pós-graduação em Saúde e Produção Animal, Universidade Federal Rural da Amazônia, Belém, PA, Brazil.; 4 Universidade Federal do Ceará, Fortaleza, CE, Brazil.; 5 Instituto de Saúde e Produção Animal, Universidade Federal Rural da Amazônia, Belém, PA, Brazil.

**Keywords:** clinical pathology, cetaceans, rhabdomyolysis, stress, physiological response, patologia clínica, cetáceos, rabdomiólise, estresse, resposta fisiológica

## Abstract

Stranded cetaceans face critical illnesses and often present with multiple co-morbidities, which are further exacerbated by the stress induced by stranding events and interactions with humans. Capture myopathy (CM) is a common condition in dolphins and other wildlife subjected to extreme stress during capture, handling, or transportation. This condition is particularly problematic in highly sensitive species such as dolphins, whose intense physiological response to stress can lead to severe complications. In this case report, we present the hematological, biochemical, and histopathological findings that contributed to the diagnosis of secondary CM in a wild Clymene dolphin, *Stenella clymene*, stranded alive on a beach in northeastern Brazil.

## Resumo

Cetáceos encalhados enfrentam doenças críticas e frequentemente apresentam múltiplas comorbidades, agravadas pelo estresse do encalhe e da interação com humanos. A miopatia de captura (MC) é uma condição comum em golfinhos e outros animais selvagens submetidos a estresse extremo durante a captura, manipulação ou transporte. Essa condição é especialmente problemática em espécies altamente sensíveis, como os golfinhos, cuja intensa resposta fisiológica ao estresse pode levar a complicações graves. Neste relato de caso, apresentamos os achados hematológicos, bioquímicos e histopatológicos que contribuíram para o diagnóstico de MC secundária em um golfinho-de-clymene (*Stenella clymene*), encontrado encalhado vivo em uma praia no nordeste do Brasil.

## Introduction

Cetacean strandings are events wherein whales, dolphins, and porpoises are washed ashore and cannot return to the water. This phenomenon can occur individually or *en masse* and affects various cetacean species worldwide (Clarke et al., 2021; Geraci & Lounsbury, 2005; Gulland et al., 2018). Although the exact causes of stranding are often unclear, potential factors include navigational errors, geomagnetic storms, climate or oceanographic events, illnesses, injuries, diseases, unique sea currents and winds, natural mortalities, and human-induced influences such as underwater noise pollution, chemical pollution, toxins, plastic ingestion, fisheries, and ship collisions (Clarke et al., 2021; Liu et al., 2022). Mass stranding is often associated with social bonding behaviors, in which healthy individuals may follow compromised pod members ashore (Mazzariol et al., 2011). However, these social dynamics may be less relevant in single-stranding events.

Efforts to rescue stranded or capture cetaceans involve coordinated responses from veterinarians, biologists, and volunteers, aiming to return the animals to their natural habitats whenever possible (Damasceno et al., 2025; Geraci & Lounsbury, 2005). Despite these efforts, the survival rate of stranded cetaceans remains a significant concern, highlighting the need for ongoing research and conservation measures to mitigate the impact of these distressing events (Gulland et al., 2018). Capture myopathy (CM) is a serious condition affecting wild animals, particularly those undergoing stressful capture, handling, or restraint procedures. In cetaceans, CM can be a critical issue because of their high sensitivity to stress. This condition involves the degeneration of muscle tissues, including skeletal and cardiac muscles, because of excessive exertion, hyperthermia, and extreme physiological stress (Câmara et al., 2020; Gulland et al., 2018; Herráez et al., 2013). Here, we describe the hematological and histopathological data that led to the diagnosis of secondary CM in a Clymene dolphin stranded alive on a beach in northeastern Brazil.

## Case report

**Figure 1:**
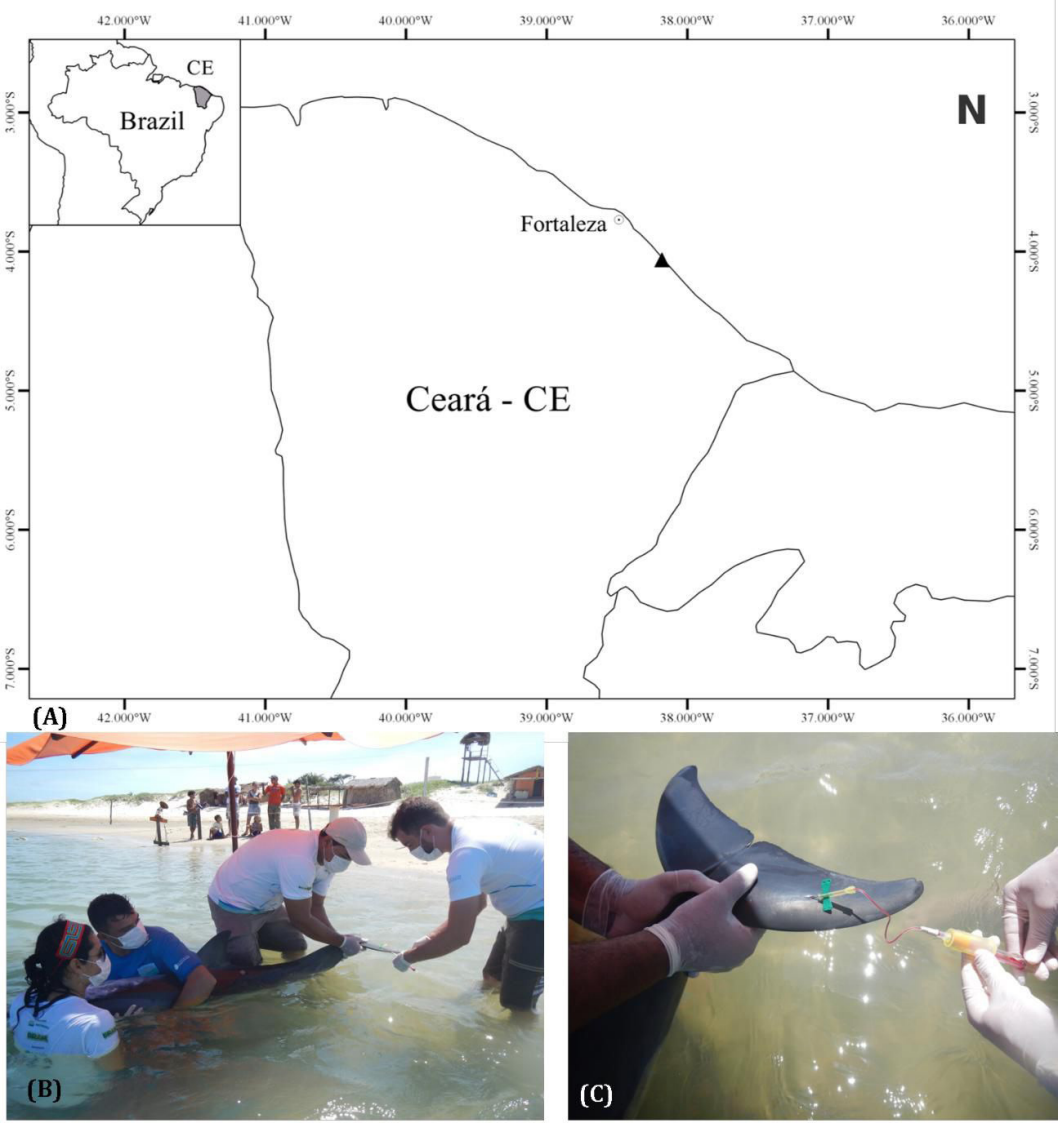
(A) Stranding location of a Clymene dolphin (*Stenella clymene*) at Águas Belas beach, Cascavel, Ceará, Brazil. (B) Physical restraint used during clinical evaluation. (C) Blood collection and administration of supportive treatment to the juvenile female.

A juvenile female Clymene dolphin (*Stenella clymene*) was found stranded alive on Águas Belas beach, Cascavel, Ceará, Brazil, on May 25, 2016, after reportedly being stranded for nearly 3 h (Figure 1A). The dolphin was in good body condition but exhibited signs of acute stress, including tachypnea, tachycardia, muscle tremors, dorsal body bending, lateralized posture, and steatorrhea (Figure 1B). Following its clinical assessment, blood samples were collected, immediately stored in appropriate vacutainer tubes, and kept in a portable cooler with ice packs at approximately 4 °C to preserve sample integrity (Figure 1C). The samples were transported to the laboratory and processed within 2 h of collection to ensure accurate analyses.

Supportive treatment was initiated based on clinical findings. It included hydration therapy with isotonic fluid, administered via gastric intubation to prevent dehydration, along with the administration of diazepam, gastric protectants (sucralfate, aluminum hydroxide, magnesium hydroxide, and simethicone), dexamethasone, nutraceuticals, prophylactic antibiotics (enrofloxacin and metronidazole), and antiparasitic drugs (ivermectin and praziquantel). In addition, the animal was provided with assisted flotation in shallow water to minimize physical exertion and facilitate breathing. All handling procedures were performed under the authorization from the System for Authorization and Information on Biodiversity/Chico Mendes Institute for Biodiversity (protocol 14104-8).

Table 1 presents the hemogram and serum biochemistry results, which were compared with the corresponding reference ranges for *Tursiops truncatus* (Bonsembiante et al., 2017) and categorized based on the degree of deviation. To standardize the classification, we defined mild deviations as deviations of 20% or less from the reference range, moderate deviations as greater than 20% up to 50%, and marked deviations as greater than 50%.

**Table 1:** Hemogram and biochemical parameters in a juvenile female *Stenella clymene* and reference ranges in *Tursiops truncatus*, according to Bossart et al. (2001) and Bonsembiante et al. (2017).

**Parameter**	**Result**	**Classification**	**Reference Range**
RBC (×10^6^/µL)	6.05	Marked increase	3.1 - 4.0
Hemoglobin (g/dL)	19.5	Moderate increase	12.7 - 15.5
Hematocrit (%)	55	Mild increase	37 – 47
MCV (fL)	90.9	Normal	11 – 127
MCHC (g/dL)	35.4	Mild increase	32 – 35
Platelets (×10^3^/µL)	406.0	Marked increase	92.0 - 217.0
Leukocytes (×10^3^/µL)	7.9	Normal	5.6 - 12.4
Neutrophil band (×10^3^/µL)	0	Normal	0
Neutrophils (×10^3^/µL)	7.27	Mild increase	2.54 - 6.14
Lymphocytes (×10^3^/µL)	0.39	Moderate decrease	0.52 - 2.42
Eosinophils (×10^3^/µL)	0	Marked decrease	0.74 - 4.53
Basophils (×10^3^/µL)	0	Normal	0 - 0.03
Monocytes (×10^3^/µL)	0.24	Normal	0.08 - 0.61
Total proteins (g/dL)	8.4	Normal	6.4 - 8.8
Albumin (g/dL)	3.6	Normal	2.9 - 3.7
Globulin (g/dL)	4.8	Normal	3.1 - 5.5
Urea (mg/dL)	77	Mild increase	45 – 72
Creatinine (mg/dL)	1.8	Normal	1.0 - 2.1
ALT (U/L)	217	Marked increase	9 – 33
AST (U/L)	850	Marked increase	133 – 318
CK (U/L)	18,070	Marked increase	100 – 250
LDH (U/L)	31,500	Marked increase	324 – 538
GGT (U/L)	82	Marked increase	17 – 31
Alkaline phosphatase (U/L)	279.2	Normal	51 – 610
Chloride (mEq/L)	124	Mild increase	108 – 118
Sodium (mEq/L)	152	Normal	151 – 158
Potassium (mEq/L)	4.2	Normal	3.2 - 4.4
Calcium (mEq/L)	7.5	Mild decrease	8.2 - 9.4
Phosphorus (mEq/L)	6.5	Normal	3.2 - 7.2

**Abbreviations:** RBC - red blood cells (morphologically normal); MCV - mean corpuscular volume; MCHC - mean corpuscular hemoglobin concentration; ALT - alanine aminotransferase; AST - aspartate aminotransferase; CK - creatine kinase; LDH - lactate dehydrogenase; GGT - gamma-glutamyl transferase. **Notes:** Platelets - platelet aggregates observed; Leukocytes - no morphological changes; Plasma - normal.

Hematological analysis revealed a marked increase in erythrocyte count, a moderate increase in hemoglobin concentration, and a mild increase in hematocrit and mean corpuscular hemoglobin concentration (MCHC). The leukogram revealed mild mature neutrophilia, moderate lymphopenia, and marked eosinopenia, whereas other hematological parameters remained within normal limits. Additionally, a marked thrombocytosis was noted, suggesting a physiological response to acute stress.

Biochemical analysis showed marked elevations in aspartate aminotransferase (AST), alanine aminotransferase (ALT), creatine kinase (CK), γ-glutamyl transferase (GGT), and lactate dehydrogenase (LDH) activities. Urea and chloride levels were mildly elevated, whereas calcium concentrations were mildly decreased. Other biochemical parameters were within reference limits. All biochemical analyses were conducted using a BIOPLUS biochemical analyzer (BIOPLUS Produtos para Laboratório Ltda., Barueri, SP, Brazil), employing colorimetric and kinetic methods using commercial Labtest kits (Labtest Diagnóstica S.A., Lagoa Santa, MG, Brazil).

Considering the high sensitivity of wild dolphins to stress and the decreased chances of survival with prolonged rehabilitation, the decision was made to release this dolphin a day after its admission. Nearly 4 days after its release, the dolphin was found stranded and exhibited signs of moderate decomposition. Necropsy revealed muscle necrosis, adhesive pleurisy, parasitic pneumonia, hepatomegaly, multifocal enteritis, and nephropathy. Parasitic findings included pulmonary nematodes (*Halocercus* sp.), gastric nematodes (*Anisakis* sp.), and peritoneal cestodes (*Monorygma grimaldii*).

**Figure 2:**
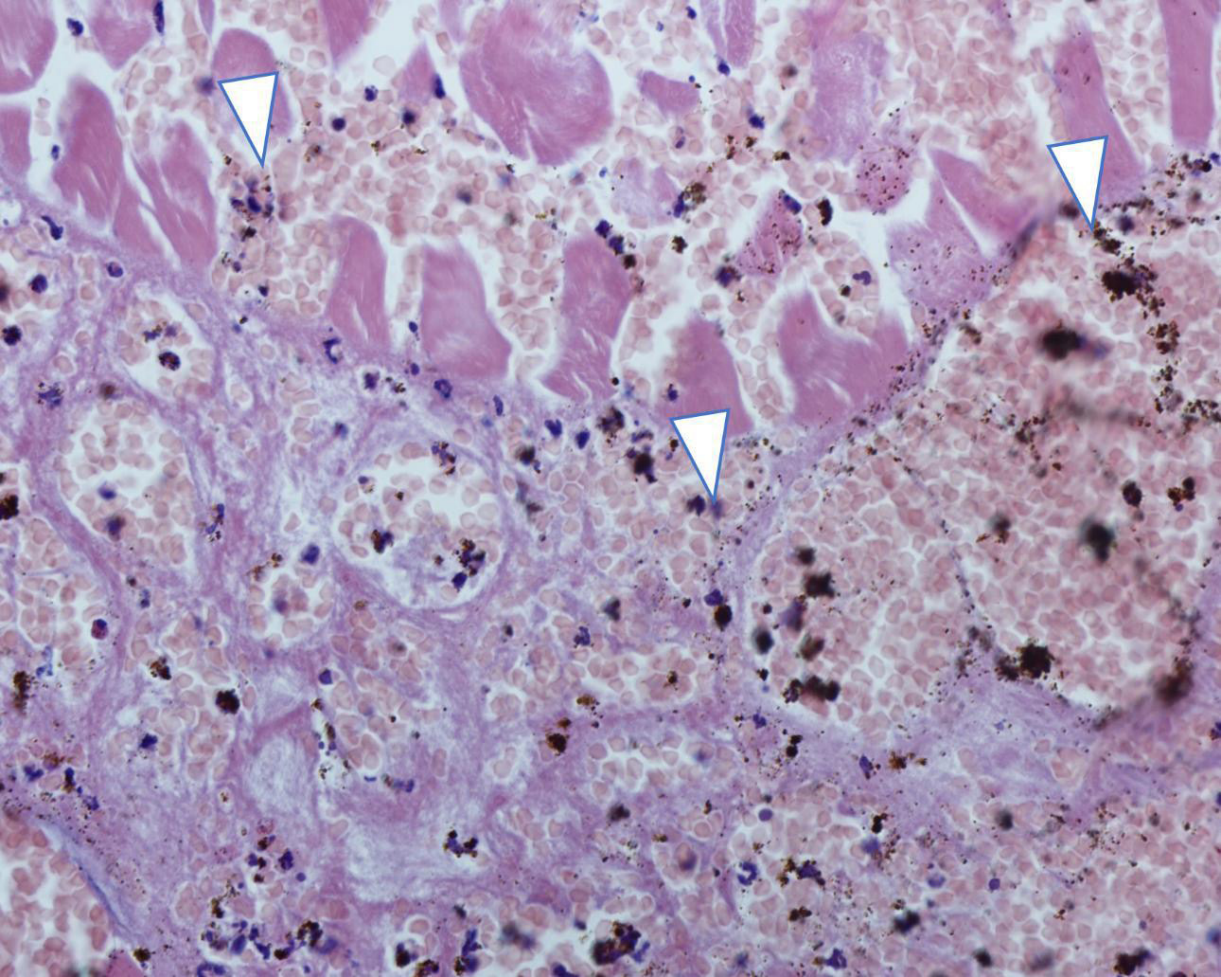
Histologic section showing rhabdomyolysis and hemorrhage in the epaxial muscles. Hematoxylin and eosin stain, 400× magnification. Note areas with disorganized fibers, dark pigment granules, and inflammatory cells; these are the key changes associated with rhabdomyolysis (head arrows).

The tissue samples were collected and fixed in 10% buffered formalin (Formalina Tampão 10%, Dinâmica Química Contemporânea Ltda., Indaiatuba, SP, Brazil) for histopathological evaluation. Severe tissue autolysis limited the analysis; however, rhabdomyolysis and hemorrhage were identified in the epaxial muscles (Figure 2). Histopathological findings also included hyperplasia of the esophageal, pulmonary, and mesenteric lymph nodes, as well as the rectal tonsils. Edema, myelitis, and moderate perivascular infiltration of lymphocytes and plasma cells were observed in the meninges, suggesting a systemic viral infection.

## Discussion

Laboratory tests play a crucial role in clinical evaluations, offering valuable insights into an animal's health. Historical data from previous physical examinations can aid in interpreting these findings, especially in suspected illness cases (Gulland et al., 2018). However, as this dolphin was stranded, no prior health records were available. The observed relative polycythemia in the erythrogram was attributed to dehydration, as it results from plasma volume loss rather than increased RBC production. This explanation aligns with the associated hematological and biochemical changes.

The hematological changes observed in the stranded juvenile *Stenella clymene* likely reflect a physiological response to stress. Leukocytosis was not present, but a mild increase in neutrophils was observed, which may indicate a stress-induced response. The release of adrenaline can mobilize leukocytes from the marginal pool into circulation and promote the release of mature neutrophils from the bone marrow, leading to transient neutrophilia (Iversen et al., 1994). However, lymphocyte and eosinophil counts were below the reference range, which may indicate stress-related lymphopenia and eosinopenia, commonly associated with corticosteroid release.

Additionally, as stress levels decreased, absolute counts of leukocytes, neutrophils, and monocytes remained within or close to normal ranges, suggesting an adaptive response over time. Monitoring the severity and hierarchy of stress can provide valuable insights into hematological and physiological changes, aiding in the assessment of stranded or capture cetaceans (Damasceno et al., 2025; Mello & Silva, 2019).

Stress leukograms, characterized by mild neutrophilia, moderate lymphopenia, and marked eosinopenia, have been documented in mass strandings, such as in a case involving 17 stranded striped dolphins (*Stenella coeruleoalba*) (Gales, 1992). The observed marked thrombocytosis was likely associated with an inflammatory response, as increased thrombopoietin is a component of the acute-phase reaction. This stress leukogram is a common physiological response in animals experiencing acute stress during stranding or handling (Gales, 1992; Iversen et al., 1994).

The increased levels of AST, CK, and LDH suggest severe muscle damage, likely due to acute stress. Although ALT is commonly associated with liver injury (Bonsembiante et al., 2017; Mello et al., 2021), it may also be mildly elevated due to muscle exertion. Increased CK activity is typically linked to skeletal or cardiac muscle injuries, but stress and resistance during capture and transport could have contributed to its elevation (Mello et al., 2021). Higher CK levels correlate with greater muscular effort.

Additionally, the significant elevations in AST, CK, and LDH, along with ALT, may indicate rhabdomyolysis and muscle hemorrhage (Lim, 2020). The increase in GGT activity supports the presence of hepatobiliary injury. Hypocalcemia, commonly associated with rhabdomyolysis, likely results from calcium sequestration in damaged muscle tissue.

Hyperchloremia and increased urea, with normal creatinine levels, suggest dehydration-related pre-renal azotemia. Elevated serum urea could also result from dehydration or other conditions, such as intestinal hemorrhage, septic shock, or cardiovascular compromise (Bonsembiante et al., 2017). Additionally, acute kidney injury cannot be ruled out, as myoglobin released from damaged muscle fibers may have nephrotoxic effects (Gulland et al., 2018; Herráez et al., 2013).

Despite signs of dehydration, total protein concentration remained within normal limits. However, the presence of steatorrhea suggests possible gastrointestinal dysfunction, though its relationship to protein metabolism remains unclear. Further diagnostic evaluations would be required to determine whether an infectious disease, metabolic disorder, or another systemic condition is contributing to these laboratory findings.

However, limited information is available on the physiological and laboratory parameters of *S. clymene* (Perrin et al., 2009). Therefore, we compared the data of *Tursiops truncatus* (Bossart et al., 2001; Bonsembiante et al., 2017) with those of the stranded individual. Deviations from these parameters indicate acute Central Nervous System (CNS) damage, likely secondary to an infectious disease that may have caused the animal to approach the shore and become stranded. The *Stenella* genus is known to be highly sensitive to stress, with some studies reporting fatal CNS damage in *S. coeruleoalba* (St. Aubin et al., 2013) and muscle damage in *S. attenuata* (Herráez et al., 2007).

Stranded cetaceans face considerable risks, including dehydration, failure in temperature regulation, and pressure-induced injuries because of their body weight. Although rescue and rehabilitation efforts may occasionally result in successful reintroduction to the ocean, many stranded cetaceans do not survive. Elucidating and addressing the causes of strandings are essential for marine conservation. In this case, the consequences of CNS damage seemed to have exacerbated the preexisting condition, leading to multiple organ failure and death. Considering that stress is a complicating factor in the care of live-stranded dolphins, thorough hematological evaluation and careful biochemistry are critical for prognosis and decision-making. Preventing CNS damage in wild dolphins requires minimizing stress during handling, ensuring adequate cooling, and using sedation to reduce physical exertion. If CNS damage is suspected in similar cases, standard veterinary approaches may include addressing electrolyte imbalances, dehydration, and potential kidney damage and providing fluid therapy, oxygen support, muscle relaxants, and anti-inflammatory medications to mitigate further complications. These interventions were partially implemented in the present case, as the animal received fluids, diazepam, gastric protectants, dexamethasone, nutraceuticals, prophylactic antibiotics (enrofloxacin and metronidazole), and antiparasitic treatments (ivermectin and praziquantel). In the case of a wild Clymene dolphin, the risks of CNS damage highlight the importance of low-stress handling protocols, particularly during rescue or research activities. This case report contributes to the knowledge and management of stranded dolphins.

## Conclusion

This case highlights the physiological impact of acute stress, dehydration, and potential CNS damage in a stranded *S. clymene*. Hematological and biochemical alterations, including stress leukogram, polycythemia, and elevated muscle enzymes, indicate significant tissue injury, likely exacerbated by rhabdomyolysis. The presence of hyperchloremia and increased urea with normal creatinine suggests dehydration-related pre-renal azotemia, though acute kidney injury cannot be ruled out. Given the lack of reference values for *S. clymene*, comparisons with *Tursiops truncatus* suggest that CNS impairment may have contributed to the stranding. Despite therapeutic interventions, the severity of the condition led to multi-organ failure and death. This case underscores the importance of low-stress handling, rapid diagnostics, and targeted treatment strategies to improve the survival of stranded cetaceans.
